# Cancer suppression by systemic inactivation of p38MAPK

**DOI:** 10.18632/oncotarget.15293

**Published:** 2017-02-11

**Authors:** Anna Brichkina, Dmitry V. Bulavin

**Affiliations:** Institute for Research on Cancer and Aging of Nice (IRCAN), INSERM, U1081-UMR CNRS 7284, University of Nice-Sophia Antipolis, Centre Antoine Lacassagne, Nice, France

**Keywords:** p38 kinase, lung cancer, hyaluronan, tumor microenvironment, stromal fibroblasts

p38MAPK (MAPK14) is a well-studied stress kinase that transmits numerous extracellular signals and is involved in multiple cellular processes. The role of p38MAPK has been rigorously investigated in many pathophysiological conditions including cancer. Originally proposed as a potential cancer suppressor [1, 2], the role of p38MAPK in tumorigenesis however remained controversial. Among the reasons is the lack of models with systemic inactivation of p38MAPK due to embryonic lethality of conventional knockouts [3, 4]. To partially overcome this problem, conditional knockouts with tissue-specific drivers were used. Initial experiments with disruption of p38MAPK in epithelial cells showed that its role in cancer cells is to suppress lung, liver and colon tumor formation in vivo [5, 6]. However, these models did not resolve the systemic functions of p38MAPK in tumorigenesis. Newly generated mouse models, with amino acid substitutions in the p38MAPK activating sites Tyr182 [7] or Pro224 [8], were embryonic viable, permitting investigation of the systemic role of p38MAPK in cancer.

**Figure 1:**
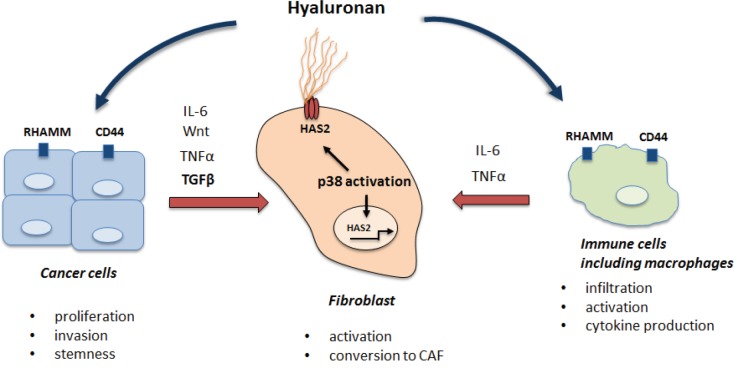
The role of p38MAPK/HA pathway in the tumor microenviroment

In contrast to expectations [5], both genetic approaches revealed that systemic downregulation of p38MAPK activity suppressed K-ras driven lung tumorigenesis [7, 8]. Further analysis showed that this effect was non-cell-autonomous, independent of p53 and acted through the regulation of the tumor microenvironment. Stromal cells play a crucial role in tumor progression by modulating ExtraCellular Matrix (ECM), secreting cytokines and soluble factors to create a cancer niche. We found that p38MAPK was crucial in early activation of lung stromal fibroblasts in response to soluble factors mostly produced by cancer cells, such as Tgf-beta (Figure 1). Similar response could also be anticipated in response to the factors secreted by inflammatory immune cells including macrophages. The relevant stromal effect regulated by p38MAPK in lung fibroblasts was the synthesis of hyaluronic acid (HA), an important component of the ECM, which is recognized as an active participant in cancer cell proliferation and invasion, as well as in angiogenesis and the inflammatory response. HA deposition is elevated in various types of cancer and the magnitude of HA accumulation in cancer stroma correlates well with the cancer aggressiveness. We showed that p38MAPK controlled the transcription of the HAS2 enzyme in stromal fibroblasts, which is critical for production of HA [7].

HA is present as high and low molecular weight (HMW-HA, LMW-HA) polymers with LMW-HA generated by hyaluronidase-dependent fragmentation of HMW-HA. An interesting paradox is that extremely high HMW-HA has been demonstrated to have an anti-tumor effect in Naked Mole Rat, a cancer-free, long lived rodent [Tian et al., 2013]. In this organism, a combination of modified HAS2 expression and insufficient degradation by hyaluronidases results in deposition of a very high HMW-HA, on average 5-6 times bigger than HMW-HA synthesized in human tissues. However, in the cancer niche, HMW-HA undergoes rapid fragmentation by hyaluronidases into LMW-HA, which is tumor-promoting. Specifically, LMW-HA interacts with the receptors, CD44 and RHAMM, to trigger cellular responses. These receptors are expressed on different cell types, and often overexpressed in cancer cells, including Cancer Stem Cells (CSC). HA-CD44 interactions in cancer cells are known to induce proliferation, maintain CSC self-renewal and to stimulate epithelial-mesenchymal transition. Apart from a direct effect on tumor cells, HA modulates the tumor microenvironment by attracting immune cells including macrophages to tumor sites, thus accelerating tumor-associated inflammation (Figure 1).

Recently, numerous clinical trials have been initiated to use p38MAPK inhibitors in combination with chemotherapy for various types of cancer (www.clinicaltrials.gov). As chemotherapy is a powerful inducer of p38MAPK activity, which in turn results in increased HAS2 activity and HA deposition, the use of either p38 or HA inhibitors could significantly dampen the cancer-promoting activity of the tumor microenvironment. Thus, from a therapeutic standpoint, the most optimal approach to target HA-mediated tumor progression could be by either blocking p38MAPK with pharmacological inhibitors, inhibiting HA synthesis (through the commercial drugs cantabilne/bilicante), targeting hyaluronidases or disrupting HA-receptor interactions. In addition, as tumor stroma is more genetically stable than highly heterogeneous cancer cells, blocking the stromal component with the p38MAPK/HA inhibitors could be suitable for various cancer types, independent of driver mutations and the tissue of origin. Regardless, a more complete understanding of the mechanisms underlying p38-driven modulation of the tumor microenvironment is warranted to develop a more effective anti-cancer treatment. In this respect, targeting a p38MAPK/HA pathway could provide a promising therapeutic approach.
